# Comparison of ChatGPT versions in informing patients with rotator cuff injuries

**DOI:** 10.1016/j.jseint.2024.04.016

**Published:** 2024-05-06

**Authors:** Ali Eray Günay, Alper Özer, Alparslan Yazıcı, Gökhan Sayer

**Affiliations:** a Department of Orthopedics and Traumatology, Kayseri City Training and Research Hospital, Kayseri, Turkey; b Department of Orthopedics and Traumatology, Develi State Hospital, Kayseri, Turkey; c Department of Orthopedics and Traumatology, Bursa City Training and Research Hospital, Bursa, Turkey

**Keywords:** Shoulder, Arthroscopy, OpenAI, Artificial intelligence, Rotator cuff, ChatGPT

## Abstract

**Background:**

The aim of this study is to evaluate whether Chat Generative Pretrained Transformer (ChatGPT) can be recommended as a resource for informing patients planning rotator cuff repairs, and to assess the differences between ChatGPT 3.5 and 4.0 versions in terms of information content and readability.

**Methods:**

In August 2023, 13 commonly asked questions by patients with rotator cuff disease were posed to ChatGPT 3.5 and ChatGPT 4 programs using different internet protocol computers by 3 experienced surgeons in rotator cuff surgery. After converting the answers of both versions into text, the quality and readability of the answers were examined.

**Results:**

The average Journal of the American Medical Association score for both versions was 0, and the average DISCERN score was 61.6. A statistically significant and strong correlation was found between ChatGPT 3.5 and 4.0 DISCERN scores. There was excellent agreement in DISCERN scores for both versions among the 3 evaluators. ChatGPT 3.5 was found to be less readable than ChatGPT 4.0.

**Conclusion:**

The information provided by the ChatGPT conversational system was evaluated as of high quality, but there were significant shortcomings in terms of reliability due to the lack of citations. Despite the ChatGPT 4.0 version having higher readability scores, both versions were considered difficult to read.

Rotator cuff tears have become increasingly important pathologies with the widespread use of magnetic resonance imaging, improved quality of life, and the popularity of arthroscopy.^4^ The prevalence is estimated to be up to 50%, especially in the geriatric population.^10^ Surgical repair may necessitate lifestyle changes in the short and long term after surgery. Currently, the most crucial step in patient satisfaction is for patients to have sufficient knowledge about their diseases.^8^ Patients and their relatives tend to research pathologies, especially those requiring surgery. The widespread use of the internet and the variety of content available have turned the internet into an excellent but difficult-to-control source of information.^7^ Patients need an easily readable, standardized source where they can obtain sufficient information.^1^^,^^7^^,^^8^

Chat Generative Pretrained Transformer (ChatGPT) is a human-like language platform based on deep learning and aggregator of information available online, developed by Open Artificial Intelligence (OpenAI, San Francisco, CA, USA) in 2018. The second version was released in 2019, and the third version in 2020. Versions 3.5 and 4.0 were introduced in 2022 and 2023, respectively, with the 4.0 version requiring a paid subscription.^17^

The aim of this study is to evaluate whether ChatGPT can be recommended as a resource for informing patients planning rotator cuff repairs, and to assess the differences between ChatGPT 3.5 and 4.0 versions in terms of information content and readability. The hypothesis of the study is that ChatGPT 4.0 will provide more comprehensive information compared to version 3.5, and both versions will be sufficient in terms of content and readability for patient information.

## Methods

### Study design

In August 2023, 13 questions commonly asked by patients with rotator cuff disease were posed to ChatGPT 3.5 and ChatGPT 4 programs (Supplementary Appendices S1 and S2) (Table I).^11^ After converting the answers into text, the quality and readability of both text responses were compared.

**Table I:** Frequently asked questions by patients with rotator cuff disease.

1. Where do they cut for rotator cuff surgery?
2. Can I drive after rotator cuff surgery?
3. What is the average recovery time for rotator cuff surgery?
4. How long does an arthroscopic shoulder surgery take?
5. What can you not do after shoulder arthroscopy?
6. How much does a rotator cuff surgery cost?
7. What happens if a torn rotator cuff goes untreated?
8. Can you wait too long for rotator cuff surgery?
9. Is rotator cuff surgery considered a major surgery?
10. Why is rotator cuff surgery so painful?
11. How long does a rotator cuff repair last?
12. Is rotator cuff surgery worth it?
13. How can I speed up recovery after rotator cuff surgery?

### Quality analysis

The evaluation was conducted by 3 experienced surgeons in rotator cuff surgery using the Journal of the American Medical Association (JAMA) comparison criteria and the DISCERN score.^2^^,^^19^ The DISCERN score was evaluated with a maximum score of 80, where a score more than 70 is classified as excellent, and a score more than 50 is classified as good (Supplementary Appendix S3).

### Readability analysis

Readability was assessed using 5 different popular reading scores: Flesch-Kincaid Reading Ease Score (FRES) and Grade Level, Simple Measure of Gobbledygook index, Coleman Liau index, and Gunning-Fox Index.^3^^,^^6^^,^^9^^,^^14^^,^^16^ FRES score ranges from 0-29 (very difficult, postgraduate) to 90-100 (very easy, fourth to fifth grade). The average of the other 4 scoring systems was also used in the evaluation.

### Statistical analysis

Data were analyzed using SPSS 22.0 (IBM Corp., Armonk, NY, USA). Inter-rater reliability analysis was performed for DISCERN results, with a value between 0.01 and 0.20 considered none, 0.21 and 0.40 considered weak, 0.41 and 0.60 considered moderate, 0.61 and 0.80 considered strong, and 0.81 and 1.00 considered perfect agreement. The similarity of scores between ChatGPT 3.5 and 4.0 responses was tested using Pearson correlation analysis.

## Results

Both versions received a JAMA score average of 0 since the source was not clearly identified. The mean DISCERN score for both ChatGPT 3.5 and 4.0 was 61.6, and the answer quality was considered good. There was no statistically significant difference in response quality between versions. A statistically significant and strong correlation was found between ChatGPT 3.5 and 4.0 DISCERN scores (r: 0.986, *P* < .001). There was excellent agreement in DISCERN scores for both versions among the 3 evaluators (Table II).

**Table II:** DISCERN and readability values of ChatGPT 3.5 and 4.0 versions and inter-rater reliability analysis.

	ChatGPT 3.5	ChatGPT 4.0
DISCERN (mean)	61.6	61.6
IRR	0.94	0.96
FRES (points)	31.0	45.6
FKGL (grade)	12.6	9.10
SMOG (grade)	14.6	13.5
Coleman Liau (grade)	15.0	12.9
Gunning-Fox Index (grade)	16.6	15.0
Grade Scale Mean (grade)	14.7	12.6

*FRES*, Flesch-Kincaid Reading Ease Score; *FKGL*, Flesch-Kincaid Grade Level; *SMOG*, Simple Measure of Gobbledygook; *ChatGPT*, Chat Generative Pretrained Transfomer; *IRR*, inter-rater reliability.

ChatGPT 3.5 was found to be less readable than ChatGPT 4.0. The FRES score for both versions was considered difficult. The average of the 4 different grade results was 14.7 for v3.5 and 12.6 for v4.0 (Table II). It was observed that the information provided at the end of medical questions recommended evaluation by an expert doctor.

## Discussion

This study found that ChatGPT contains good-quality information for informing patients with rotator cuff injuries but does not provide reliable citations. While there was no difference in quality between versions, the paid 4.0 version was able to convey information in more understandable English.

The scientific content and quality of the answers were evaluated using the DISCERN scoring system, which examines the content quality of materials used to inform patients and is funded by the National Health Service Executive Research and Development Program. It includes 16 questions scored on a scale of 1-5, and materials can receive a total score between 16 and 80. A score above 70 is considered excellent, and above 50 is considered good. Previous evaluations of online content quality for shoulder patients have shown varying results. Dalton et al^5^ in 2015 found a DISCERN score of 39.5, indicating poor information content for internet sources. Lawson et al^15^ in 2016 found the highest DISCERN score on academic sites to be 51, with a general website DISCERN average of 44. In 2023, Hurley et al^12^ found the average DISCERN score to be 60 in their study examining artificial intelligence’s information about shoulder instability. This indicates an improvement in information content quality over time, especially with the use of artificial intelligence. Our study suggests that ChatGPT answers have a higher DISCERN value compared to other online information sources in the literature. Due to the self-improving nature of artificial intelligence, it is believed that this value will increase even further in the future.

Visual or audio-supported narratives in informing patients may be more beneficial in conveying more information in an understandable language. In a study by Jessen et al^13^ in 2022 comparing information about subacromial impingement YouTube (48 videos) and Google search engine (58 website) information, DISCERN score results were 33.1 vs. 48.5, respectively. Although DISCERN scores were low with visually supported narratives, it is believed that better results can be achieved in the future with visually supported narratives with artificial intelligence.

The self-improving nature of artificial intelligence provides great convenience in accessing information, but concerns about the source of information and copyright issues are growing problems as its use grows. Due to this lack of transparency, ChatGPT’s responses are viewed with suspicion. In this study, the artificial intelligence chatbot never referred to any source material and received “0 point” from the JAMA comparison criteria. Therefore, although it is recommended that patients obtain information from ChatGPT, they should be informed that its reliability is low. In addition, ChatGPT directed patients to orthopedic specialists in all answers for access to the main information, as it does not have much confidence in itself.

Studies have found some high-quality medical information on the internet, but these sources generally require a very high reading level for an ordinary person, are boring, and hard to read. Furthermore, there has been no relationship found between readability and the quality of information. In 2016, Lawson et al^15^ found a FRES score of 50.17, equivalent to an average note level of 10.98. However, no relationship was found between the readability of the website and the DISCERN score. Dalton et al^5^ evaluated 59 websites returned after searching for “rotator cuff tear” on popular internet search engines. They found that the average reading note was above 9.9. Since the average reading level among US adults is not higher than the eighth-grade level, the National Institutes of Health, the Centers for Disease Control and Prevention, and the American Medical Association recommend that patient health materials be written at or below the sixth-grade reading level.^18^

### Limitations and future expectations

The limitations of this study include the inability to compare with visual informants and the evaluation of a single artificial intelligence program based on a single response. In the future, studies can be planned to evaluate responses obtained from different internet protocol computers at different times and compare these results with visual informants (YouTube, etc.).

## Conclusion

ChatGPT contains good-quality information for informing patients with rotator cuff injuries but does not provide reliable open source. While there was no difference in quality between versions, the paid 4.0 version was able to convey information in more understandable English. As it stands, ChatGPT is seen as a source for shoulder patients to access information, and the self-improving nature of artificial intelligence suggests that it will become even more useful in the future.

## Disclaimers:

Funding: No funding was disclosed by the authors.

Conflicts of interest: The authors, their immediate families, and any research foundation with which they are affiliated have not received any financial payments or other benefits from any commercial entity related to the subject of this article.

## Declaration of generative AI and AI-assisted technologies in the writing process

During the preparation of this work, the authors used ChatGPT (OpenAI, San Francisco, CA, USA) to evaluate the answers of this program. After using this tool/service, the authors reviewed and edited the content as needed and took full responsibility for the content of the publication.
