# Supplementary material for: Comparison of ChatGPT versions in informing patients with rotator cuff injuries
Source: JSES Int. 2024 May 6;8(5):1016–8. doi: 10.1016/j.jseint.2024.04.016 (PMC11401580; doi:10.1016/j.jseint.2024.04.016)
Supplement: Supplementary Appendix S1 [file mmc1.docx]

| D-Q1 | Are the aims clear ? |
| --- | --- |
| D-Q2 | Does it achieve its aims ? |
| D-Q3 | Is it relevant ? |
| D-Q4 | Is it clear what source of information were used to compile the publication (other than the author or producer)? |
| D-Q5 | Is it clear when the information used or reported in the publication was produced ? |
| D-Q6 | Is it balanced and unbiased ? |
| D-Q7 | Does it provide details of additional sources of support and information ? |
| D-Q8 | Does it refer to areas of uncertainty ? |
| D-Q9 | Does it describe how each treatment works ? |
| D-Q10 | Does it describe the benefits of each treatment ? |
| D-Q11 | Does it describe the risks of each treatment ? |
| D-Q12 | Does it describe what would happen if no treatment is used ? |
| D-Q13 | Does it describe how the treatment choices avect overall quality of life ? |
| D-Q14 | Is it clear that there may be more than one possible treatment choice ? |
| D-Q15 | Does it provide support for shared desicion-making ? |
| D-Q16 | Based on the answers to all of the above questions, rate the overall quality of the publication as a source of information about treatment choices? |

**Table 1.** Discern Scoring System (The text is evaluated by answering the following questions. For each question, points are given as 5 is the best - 1 is the worst. In total, the text receives a score between 16 and 80)

**Table 2.** Journal of American Medical Association (JAMA) benchmark criteria (The text is evaluated against the following four criteria. Each criterion met receives 1 point, with a maximum of 4 points in total.)

| **Authorship** | Authors and contributors, their affiliations, and relevant credentials should be provided |
| --- | --- |
| **Attribution** | References and sources for all content should be listed clearly, and all relevant copyright information should be noted |
| **Disclosure** | Website “ownership” should be prominently and fully disclosed, as should any sponsorship, advertising, underwriting, commercial funding arrangements or support, or potential conflicts of interest |
| **Currency** | Dates when content was posted and updated should be indicated |
