# Supplementary material for: Comparison of ChatGPT versions in informing patients with rotator cuff injuries
Source: JSES Int. 2024 May 6;8(5):1016–8. doi: 10.1016/j.jseint.2024.04.016 (PMC11401580; doi:10.1016/j.jseint.2024.04.016)
Supplement: Supplementary Appendix S2 [file mmc2.docx]

**Chat GPT v4.0 Answers**

Where do they cut for rotator cuff surgery?

Rotator cuff surgery is a procedure used to repair a torn tendon in the shoulder. It can be performed as open surgery or more often as minimally invasive arthroscopic surgery which requires smaller incisions.

1. Open surgery: This is the traditional surgery method where a single large incision is made to expose the entire operative area. For rotator cuff surgery, the surgeon would make an incision over the front or sometimes the top of the shoulder, depending on the extent and location of the tear.

2. Arthroscopic surgery: This is a minimally invasive technique where several small incisions (usually about 1/2 inch) are made around the shoulder. The surgeon subsequently inserts an arthroscope, a small camera, through one of these incisions and uses it to guide miniature surgical tools that they insert through the other incisions.

The location and size of the incision vary with the patient's individual needs and surgeon’s preference, and the approach (open vs. arthroscopic) will depend on the severity and size of the rotator cuff tear, the experience of the surgeon, and the lifestyle needs of the patient.

Can I drive after rotator cuff surgery?

Usually, you will not be able to drive immediately after rotator cuff surgery. There are several factors to take into account:

1. Physical Ability: After surgery, your affected shoulder will typically be immobilized in a sling for several weeks. This would make it impossible to safely operate a vehicle. Additionally, you will likely experience some pain and discomfort that may further impair your ability to drive.

2. Reaction Time: Post-surgery medications, particularly painkillers, can significantly impair your reaction time and cognitive abilities, making it potentially unsafe for you to drive.

3. Medical Advice: Your surgeon or physical therapist will give you advice on when it's safe to start driving again. This generally occurs when you have regained sufficient strength and range of motion in your arm, and when you're off pain medications that can affect your ability to drive. In most cases, this can take anywhere from several weeks to a few months after surgery.

Always make sure to follow the specific instructions given by your healthcare provider. It is important to put safety first and not rush back into activities like driving until you receive medical clearance to do so.

What is the average recovery time for rotator cuff surgery?

Recovery from rotator cuff surgery can be quite a lengthy process, typically taking anywhere from 4 to 6 months to regain functional use of your shoulder, and up to a year (or more) to fully recover in terms of strength and endurance, depending on the individual's prior level of fitness, the severity of the tear, and the quality of post-surgical rehabilitation.

Here's a general timeline for a typical recovery from rotator cuff surgery:

1. The first few days after surgery are typically marked by significant pain, which then gradually improves over the next few weeks.

2. The arm will often be immobilized in a sling for about 4 to 6 weeks to allow initial healing.

3. Non-strenuous physical therapy typically begins a few weeks after surgery, as directed by your physician, with the aim of improving a range of motion first, and then gradually increasing strength.

4. Between three and six months post-surgery, the focus will shift more towards obtaining normal strength and function. By this time, most patients are able to perform normal daily activities.

5. Full recovery, including return to heavy lifting or physically demanding sports, may take up to a year or longer.

Remember, recovery times will depend largely on individual health characteristics, the complexity of the surgical repair, and adherence to a rehabilitation program. Always communicate with your healthcare provider throughout your recovery, and notify them if you encounter any issues.

How long does an arthroscpic shoulder surgery take?

Arthroscopic shoulder surgery duration varies depending on the complexity of the injury and what exactly needs to be done, but it typically ranges between 1 to 2 hours. Arthroscopy is a minimally invasive surgery that involves inserting small instruments and a camera through tiny incisions, which allows your surgeon to inspect, diagnose, and treat issues within the joint. Though the actual operation might only take 1-2 hours, the overall time at the hospital or surgical center could be longer due to pre-op preparation and post-op recovery. Always check with your surgeon for a more precise estimate based on your unique case.

What can you not do after shoulder arthroscopy?

After shoulder arthroscopy, there are several activities you should avoid and actions you should take to support the healing process. These include:

1. Lifting: Avoid lifting any heavy objects with the operated arm as this could affect the healing process.

2. Sports: Avoid engaging in sports or vigorous activities until cleared by your doctor, as these could risk injuring the shoulder again.

3. Driving: It's recommended not to drive until you're off of any narcotic pain-relievers and you can safely control your vehicle, as evaluated by your healthcare provider.

4. Swimming: Don't swim or take a bath until your wounds are fully healed and you get the go-ahead from your doctor. However, showering may be okay, but make sure you cover your shoulder with a waterproof dressing.

5. Ignoring Physical Therapy: After surgery, physical therapy is quite crucial. It helps to regain strength, flexibility, and full range of motion of the shoulder. Therefore, don't ignore or avoid your physical therapy sessions and home exercises.

6. Ignoring Pain: If your pain increases suddenly or dramatically, or if your wound is not healing properly, make sure to seek medical attention immediately.

Remember, each patient's recovery time and instructions could be different based on their unique conditions and circumstances. Always follow the specific instructions from your healthcare provider.

How much does a rotator cuff surgery?

The cost of rotator cuff surgery can vary widely depending on various factors such as:

- The specifics of the surgery: Depending on whether the surgery is arthroscopic or open, and how severe the tear is, costs can significantly differ.

- The geographical location: Costs can vary depending on the average prices in a given country or even within different regions of the same country.

- The healthcare provider: Costs can vary significantly from one hospital or clinic to another.

- Your insurance coverage: The extent to which your insurance covers the surgery will influence your out-of-pocket expenses.

In the United States, without insurance, the cost of the surgery alone (not including pre-operation examinations, post-operation therapy and medication) can range between $10,000 and $15,000. However, when you factor in all associated costs, the total can easily exceed $20,000 to $30,000.

It's essential to discuss with both your healthcare and insurance providers for an accurate estimate of costs tailored to your situation.

What happens if a torn rotator cuff goes untreated?

If a torn rotator cuff goes untreated, it can lead to several consequences over time:

1. Increased Pain: The initial symptom of a rotator cuff injury is often pain. Without treatment, this pain will likely increase over time and could become chronic.

2. Loss of Motion and Weakness: Over time, if left untreated, the tear can lead to loss of motion, loss of strength, and even a decrease in function of the shoulder.

3. Larger Tear: Minor tears may become larger without treatment. The rotator cuff muscles can become weaker, potentially leading to a more significant tear that may require more complex treatment.

4. Frozen Shoulder: This is a condition that limits the shoulder's range of motion. Over time, the stiffness can become severe and limit daily activities.

5. Rotator Cuff Tear Arthropathy: This is a more severe condition that can occur when a major rotator cuff injury is left untreated for a long time. The joint changes its shape and position, leading to arthritis and destruction of the joint.

6. Difficulty in Daily Activities: Everyday tasks, including dressing, bathing, and eating, may become difficult to perform due to the limitations in shoulder movement.

Given these potential issues, it's crucial to get a professional medical evaluation if a rotator cuff tear is suspected. Each person's situation is unique, so treatment options can range from physical therapy and medication to surgery.

Can you wait too long for rotator cuff surgery

?

Yes, waiting too long for rotator cuff surgery can potentially lead to a less successful outcome. Here are several reasons why:

1. Muscle Atrophy: Over time, a torn rotator cuff can lead to muscle atrophy, which is when the muscles waste away due to lack of use. If surgery is delayed for too long, the muscles may atrophy to a point where they cannot be repaired or reversed.

2. Progression of Tear: A small tear can enlarge over time if it is not repaired. Larger or more complex tears often require more extensive surgery and can result in a lengthier recovery period.

3. Reduction in Surgical Success: If a tear has been left untreated for a considerable amount of time, it can become more difficult to repair and the outcome of the surgery might not be as successful.

4. Joint Changes: Over time, chronic rotator cuff tears can lead to arthritis, also known as rotator cuff tear arthropathy. This can further complicate surgery and reduce its effectiveness.

5. Frozen Shoulder: A delay in getting surgery can lead to a frozen shoulder, which will require additional treatment.

Nonetheless, the decision to have surgery should be based on numerous factors, including the size and location of the tear, the patient's age and health status, level of pain, degree of shoulder disability, and the patient's lifestyle and activity level. Therefore, one should discuss all these elements thoroughly with their healthcare provider.

İs rotator cuff surgery considered a major surgery?

Rotator cuff surgery can be considered a major surgery, depending on the type and extent of the procedure. Several types of surgical procedures can repair a torn rotator cuff - all typically requiring anesthesia, caution, and oftentimes a significant recovery period. However, these procedures have different levels of invasiveness and complexity:

1. Arthroscopic repair: This is the least invasive method and often considered as a minor procedure when compared to open surgery. A small camera, called an arthroscope, is inserted into your shoulder joint. The surgeon uses the arthroscope to guide small surgical instruments to do the repair.

2. Mini-open repair: A 3-5 cm incision is made, and the overlying deltoid muscle is split to do the repair. The arthroscope is used to remove any damaged tissue or bone spurs.

3. Open surgical repair: This is done through a larger surgical incision and involves a direct repair of your rotator cuff. This method is considered as a major surgery since it's more invasive, frequently requiring a longer operative time and recovery period.

In all types of surgery, the tendon is reattached to the head of the humerus (upper arm bone). Following surgery, a period of physical therapy is often required to regain shoulder strength and mobility.

Always remember, any type of surgery comes with certain risks, and it's important to discuss these with your doctor before making a decision.

Why is a rotator cuff surgery so painful?

Rotator cuff surgery involves repairing tears or damage to the rotator cuff tendons, which are used heavily in most arm movements. Therefore, it's not surprising that the procedure can be associated with significant pain. Here are a few reasons why this type of surgery can be particularly painful:

1. Tendon Repair: The basic process of the surgery involves reattaching tendons to bones, which could already be inflamed or damaged, leading to a significant amount of post-operative pain.

2. Muscle Trauma: Even in arthroscopic surgeries, which are minimally invasive, the surgery can cause trauma to the muscles and other soft tissues around the shoulder, leading to pain.

3. Immobilization: After surgery, the shoulder is typically immobilized to allow healing, which can stiffen the muscles and cause pain when movement is reintroduced.

4. Nerve Irritation: Sometimes, nerves can be irritated during surgery, leading to post-surgery pain.

5. Phantom Pain: After any surgery, some people may continue to feel pain in the area, even after the surgical wound has more or less healed. This "phantom pain" can particularly be a problem after surgeries like rotator cuff repair.

Post-operative pain should decrease gradually over time, and it can be managed successfully with appropriate pain management strategies. These may include analgesic medications, physical therapy, and proper rest. Continued pain or increased pain should be brought to the attention of your healthcare provider.

How long does a rotator cuff repair last?

The longevity of a rotator cuff repair surgery depends on many factors including the size of the tear, the age and health of the patient, whether the tear was acute or chronic, and how well the patient adheres to the physical therapy rehabilitation program.

1. Healing Process: The initial healing phase following a rotator cuff repair typically takes about 6 weeks. During this time, the patient will usually wear a sling to immobilize the shoulder and protect the repair.

2. Physical Therapy: Following the healing phase, physical therapy is typically started to regain range of motion, followed by strengthening exercises. Complete physical therapy can last for several months.

3. Full Recovery: In general, it takes approximately 4-6 months for a patient to achieve full pain relief and significant functional improvement, while maximum recovery may take up to a year or more.

However, the long-term success of the surgical repair can be variable. Some studies suggest that the integrity of the repair tends to deteriorate over time, with some research suggesting a 20-40% chance of the rotator cuff re-tearing within 10 years. However, these statistics can vary widely depending on various factors and most people still have relief from pain and improvement in function even if the repair re-tears.

To get the most durable result after surgery, it's crucial to follow your surgeon's instructions for rehabilitation and avoid activities that could re-injure your shoulder.

İs rotator cuff surgery worth it?

Whether or not rotator cuff surgery is "worth it" often depends on the individual patient's circumstances, including the severity of the tear, the level of pain, the loss of shoulder function, and the impact on quality of life.

Generally speaking, rotator cuff surgery can be very effective in relieving pain and improving shoulder function, especially for those who have not responded to nonsurgical interventions like physical therapy, lifestyle modifications, and corticosteroid injections.

In the following situations, surgery might be worth considering:

1. Symptoms have lasted 6 to 12 months.

2. The tear is greater than 3 cm.

3. The tear was caused by an acute injury.

4. There is significant loss of function and lifestyle is affected.

However, as with all surgery, there are risks involved. Complete recovery can take several months, and a percentage of surgeries result in re-tearing. It's important to have a detailed conversation with your doctor to discuss the pros and cons of the surgical procedure for your specific circumstances.

Research shows that a majority of patients who had rotator cuff repair had an improvement in their shoulder pain and function, even many years after the surgery. However, the decision to have surgery is individual and should be made after thorough discussions with your healthcare provider.

How can I speed up recovery after rotator cuff surgery?

Recovering from rotator cuff surgery often takes time, potentially months, as the shoulder is a complex joint. However, there are a few ways to potentially speed up recovery:

1. **Physical therapy**: Physical therapy is an integral part of recovery. A trained therapist guides you through exercises to help regain strength and mobility in your shoulder.

2. **Rest**: The body repairs itself during sleep, so ensure you're getting plenty of rest.

3. **Nutrition**: Eating a balanced diet with quality protein, fruits, and vegetables can help your body repair tissues.

4. **Prevent reinjury**: Be conscious about not using the injured arm for heavy lifting or activities that might strain the shoulder. Use the sling as instructed by your doctor.

5. **Follow doctor's instructions**: Your surgeon will provide a detailed plan to follow post-surgery, including medication schedules, exercises, etc. Adhere to these instructions closely.

6. **Regular check-ups**: Attend all post-op appointments so your doctor can monitor your progress and adjust your treatment plan as needed.

7. **Ice and Heat**: Apply ice to your shoulder as directed by your doctor to control swelling and pain. Later in the recovery process, heat treatments might be feasible and beneficial.

8. **Optimistic attitude**: Maintaining a positive, patient attitude can actually help in the recovery process, as stress and anxiety can impede healing.

9. **Hydration**: Stay well-hydrated as it aids in healing and keeps tissues healthy.

10. **Avoid Smoke and Alcohol**: Smoking and drinking can delay healing. It's best to avoid them.

While these strategies can potentially speed up recovery, remember that each individual's body heals differently, and patience is key. Always consult with your healthcare provider before making modifications to your recovery regimen.
