# Supplementary material for: Comparison of ChatGPT versions in informing patients with rotator cuff injuries
Source: JSES Int. 2024 May 6;8(5):1016–8. doi: 10.1016/j.jseint.2024.04.016 (PMC11401580; doi:10.1016/j.jseint.2024.04.016)
Supplement: Supplementary Appendix S3 [file mmc3.docx]

**Chat GPT v3.5 Answers**

Where do they cut for rotator cuff surgery ?

Rotator cuff surgery involves repairing or reattaching torn tendons in the shoulder, and the specific location of the surgical incision can vary based on the surgical technique used and the surgeon's preference. However, there are generally two main approaches for rotator cuff surgery:

1. **Arthroscopic Surgery:** This is a minimally invasive approach where the surgeon makes small incisions (usually around 1/4 to 1/2 inch each) around the shoulder joint. A tiny camera (arthroscope) is inserted through one of the incisions to provide visualization of the inside of the joint. Surgical instruments are inserted through the other incisions to repair the rotator cuff. Arthroscopic surgery is less invasive and generally leads to quicker recovery and less scarring.
2. **Open Surgery:** In some cases, a larger incision (3 to 6 inches) may be made directly over the shoulder joint. This approach provides the surgeon with direct access to the torn rotator cuff tendons. Open surgery may be necessary for more complex or extensive tears.

The exact location of the incision can vary within the general guidelines mentioned above, and your surgeon will determine the most appropriate approach based on your specific condition and needs. It's important to discuss the surgical technique, potential risks, benefits, and recovery expectations with your orthopedic surgeon before undergoing the procedure.

Can I drive after rotator cuff surgery ?

After rotator cuff surgery, your ability to drive will depend on several factors, including the type of surgery you had, the shoulder involved, your level of pain, your range of motion, and the recommendations of your surgeon. Here are some general guidelines:

1. **Right Shoulder Surgery (for Right-Handed Drivers):** If you had surgery on your right shoulder and you are right-handed, driving may be more challenging and potentially unsafe during the early stages of recovery. Operating the steering wheel and gear shift with your affected arm may not be possible or comfortable. You should avoid driving until you have regained sufficient strength, range of motion, and control in your right arm.
2. **Left Shoulder Surgery (for Right-Handed Drivers):** If you had surgery on your left shoulder and you are right-handed, you may be able to resume driving sooner, especially if you drive an automatic transmission vehicle. However, you should still consult with your surgeon and follow their recommendations.
3. **Driving and Non-Dominant Arm Surgery:** If the surgery was performed on your non-dominant arm (the arm you use less frequently), driving may be possible earlier, especially if you have an automatic transmission vehicle. However, you should still discuss this with your surgeon.

It's important to note that in the early stages of recovery, your arm may be immobilized or in a sling, and you may be experiencing pain, limited range of motion, and weakness. These factors can impact your ability to control a vehicle safely.

Before resuming driving, you should follow your surgeon's post-operative instructions closely and consider the following:

- **Pain and Comfort:** You should be able to control the steering wheel, gear shift (if applicable), and other controls comfortably without significant pain.
- **Range of Motion:** You should have sufficient range of motion in your shoulder to perform necessary driving maneuvers safely.
- **Strength:** Your arm should have enough strength to operate the controls effectively.
- **Medications:** Some pain medications can affect your alertness and reaction times, so make sure you understand the effects of any medications you are taking.
- **Safety:** Always prioritize safety. If you have any doubts about your ability to drive safely, it's better to wait until you have fully recovered.

It's essential to have a discussion with your surgeon about when it's safe for you to resume driving after rotator cuff surgery. They will provide personalized guidance based on your specific situation and the progress of your recovery.

What is the average recovery time for rotator cuff surgery?

The recovery time for rotator cuff surgery can vary widely depending on factors such as the severity of the tear, the surgical technique used, your overall health, your commitment to rehabilitation, and how well you follow your surgeon's post-operative instructions. However, here is a general timeline for the different phases of recovery:

1. **Immediate Post-Operative Period (0-6 Weeks):** During the first few weeks after surgery, your shoulder will likely be immobilized in a sling to protect the repair and allow initial healing. You will likely experience pain, swelling, and limited range of motion. Physical therapy may begin early to focus on gentle range-of-motion exercises and maintaining joint mobility. Light activities, such as writing or typing, may be possible.
2. **Early Rehabilitation (6-12 Weeks):** As healing progresses, your surgeon and physical therapist will gradually increase the intensity of your exercises. Active range-of-motion exercises and strengthening exercises will become more important. You may be able to start using your arm for daily activities with caution. Your surgeon's guidance and the therapist's plan will be crucial during this phase.
3. **Intermediate Phase (3-6 Months):** This phase involves continued progression of strengthening exercises and functional activities. Your range of motion and strength should gradually improve. You may be able to engage in more complex daily tasks, but you should still avoid heavy lifting or strenuous activities.
4. **Advanced Rehabilitation (6-9 Months and Beyond):** During this phase, you'll work on further increasing your strength and gradually returning to more demanding activities. Your physical therapist will tailor the exercises to your specific goals, which might include returning to sports or other physically demanding tasks. Full recovery can take up to a year or more in some cases.

It's important to note that individual recovery times can vary. Some people may progress more quickly, while others may need more time. Adhering to your rehabilitation program, attending physical therapy sessions, and following your surgeon's instructions are essential for a successful recovery.

Keep in mind that returning to full function and normal activities can take several months, and it may take longer for some people, especially for those with more extensive tears or complications. It's crucial to have realistic expectations and be patient throughout the recovery process. Your surgeon will monitor your progress and adjust your rehabilitation plan accordingly.

What is the average recovery time for rotator cuff surgery?

The recovery time for rotator cuff surgery can vary widely depending on factors such as the severity of the tear, the surgical technique used, your overall health, your commitment to rehabilitation, and how well you follow your surgeon's post-operative instructions. However, here is a general timeline for the different phases of recovery:

1. **Immediate Post-Operative Period (0-6 Weeks):** During the first few weeks after surgery, your shoulder will likely be immobilized in a sling to protect the repair and allow initial healing. You will likely experience pain, swelling, and limited range of motion. Physical therapy may begin early to focus on gentle range-of-motion exercises and maintaining joint mobility. Light activities, such as writing or typing, may be possible.
2. **Early Rehabilitation (6-12 Weeks):** As healing progresses, your surgeon and physical therapist will gradually increase the intensity of your exercises. Active range-of-motion exercises and strengthening exercises will become more important. You may be able to start using your arm for daily activities with caution. Your surgeon's guidance and the therapist's plan will be crucial during this phase.
3. **Intermediate Phase (3-6 Months):** This phase involves continued progression of strengthening exercises and functional activities. Your range of motion and strength should gradually improve. You may be able to engage in more complex daily tasks, but you should still avoid heavy lifting or strenuous activities.
4. **Advanced Rehabilitation (6-9 Months and Beyond):** During this phase, you'll work on further increasing your strength and gradually returning to more demanding activities. Your physical therapist will tailor the exercises to your specific goals, which might include returning to sports or other physically demanding tasks. Full recovery can take up to a year or more in some cases.

It's important to note that individual recovery times can vary. Some people may progress more quickly, while others may need more time. Adhering to your rehabilitation program, attending physical therapy sessions, and following your surgeon's instructions are essential for a successful recovery.

Keep in mind that returning to full function and normal activities can take several months, and it may take longer for some people, especially for those with more extensive tears or complications. It's crucial to have realistic expectations and be patient throughout the recovery process. Your surgeon will monitor your progress and adjust your rehabilitation plan accordingly.

How long does an arthroscopic shoulder surgery take ?

The duration of arthroscopic shoulder surgery can vary depending on several factors, including the specific procedure being performed, the complexity of the case, the surgeon's experience, and any unexpected findings during the surgery. On average, arthroscopic shoulder surgery typically takes anywhere from 1 to 2 hours. However, it's important to note that this is a general estimate and the actual time may be shorter or longer.

Here are a few examples of common arthroscopic shoulder procedures and their approximate durations:

1. **Arthroscopic Rotator Cuff Repair:** This procedure involves repairing a torn rotator cuff tendon. The surgery can take around 1 to 2 hours, depending on the size and complexity of the tear.
2. **Arthroscopic Labral Repair:** Labral tears, such as Bankart or SLAP tears, can be repaired arthroscopically. The surgery typically takes around 1 to 2 hours.
3. **Arthroscopic Subacromial Decompression:** This procedure involves removing bone or tissue to create more space in the subacromial space, reducing impingement of the rotator cuff tendons. It usually takes about 1 hour.
4. **Arthroscopic Capsular Release:** This procedure is performed to address frozen shoulder (adhesive capsulitis) by releasing tight or scarred tissue in the shoulder joint capsule. The surgery may take around 1 to 2 hours.

It's important to have a discussion with your surgeon before the surgery to get a better understanding of the expected duration for your specific procedure. Additionally, keep in mind that the time spent in the operating room is just one part of the overall surgical process. Pre-operative preparations, anesthesia administration, and post-operative recovery and monitoring are also factors that contribute to the total time you will spend at the medical facility on the day of the surgery.

What can you not do after shoulder arthroscopy?

After shoulder arthroscopy, there are several precautions and restrictions that you should follow to promote proper healing and minimize the risk of complications. Your surgeon and physical therapist will provide you with specific instructions tailored to your individual case, but here are some common guidelines for what you should generally avoid after shoulder arthroscopy:

1. **Immobilization and Sling Use:** You will likely be instructed to wear a sling to support and protect your shoulder immediately after surgery. Follow your surgeon's guidance on how long you should wear the sling and whether you can remove it for certain activities.
2. **Overexertion and Heavy Lifting:** Avoid lifting heavy objects or engaging in strenuous activities with your operated arm for the initial weeks following surgery. Your surgeon will provide guidelines on when you can gradually start increasing the weight you lift.
3. **Sudden Movements:** Avoid sudden, jerky movements or motions that could strain or stress your healing shoulder. This includes reaching behind your back, making quick overhead movements, or forcefully pushing or pulling.
4. **Driving:** Driving may be restricted for a period of time, especially if your right shoulder (for right-handed drivers) or left shoulder (for left-handed drivers) was operated on. Your surgeon will advise you on when it is safe to resume driving.
5. **Repetitive Movements:** Certain repetitive or overhead activities should be avoided during the early stages of recovery. These might include activities like swimming, throwing, or playing sports.
6. **Sleeping Position:** It may be recommended that you avoid sleeping on the operated shoulder for a period of time to prevent putting unnecessary stress on the surgical site.
7. **Physical Activities:** Activities that put significant strain on the shoulder joint, such as weightlifting, contact sports, or intense exercise, should be avoided during the initial stages of recovery. You'll need to gradually reintroduce these activities under the guidance of your medical team.
8. **Medications and Alcohol:** Follow your surgeon's recommendations regarding pain medications and avoid alcohol while taking prescribed medications.
9. **Heat and Ice:** Use of heat and ice on the surgical site should be done as directed by your surgeon or physical therapist to manage pain and swelling.
10. **Skipping Rehabilitation:** Physical therapy is a crucial part of your recovery. Skipping or neglecting your prescribed exercises and therapy sessions can hinder your progress.

It's important to note that every individual's recovery is unique, and your surgeon will provide you with personalized instructions based on your specific procedure and needs. Always follow your surgeon's advice closely and communicate any concerns or changes in your condition during the recovery process.

How much does a rotator cuff surgery cost?

The cost of rotator cuff surgery can vary widely based on factors such as the specific procedure performed, the location of the medical facility, the surgeon's experience, the type of anesthesia used, the length of hospital stay (if required), and any potential complications that may arise during or after the surgery. Additionally, the cost can vary depending on whether you have health insurance coverage and the terms of your insurance policy.

In the United States, the cost of rotator cuff surgery (including hospital fees, surgeon's fees, anesthesia, and other related expenses) can range from several thousand to tens of thousands of dollars. Without insurance, the cost can be substantially higher. If you have health insurance, your out-of-pocket expenses will depend on factors such as your deductible, co-payments, and coverage limits.

It's important to contact your insurance provider and the medical facility where the surgery will be performed to obtain a more accurate estimate of the cost based on your specific situation. They can provide you with information about coverage, pre-authorization requirements, and any potential financial obligations you may have.

Keep in mind that medical costs can vary significantly between countries and regions, so if you are located outside of the United States, the cost of rotator cuff surgery may be different.

Before undergoing surgery, it's essential to have a clear understanding of the potential costs and to discuss financial matters with your healthcare providers and insurance company.

What happens if a torn rotator cuff goes untreated?

If a torn rotator cuff goes untreated, it can lead to a variety of potential complications and worsen over time. The severity of these complications can vary based on the size of the tear, your age, activity level, and overall shoulder health. Here are some of the possible consequences of leaving a torn rotator cuff untreated:

1. **Pain and Discomfort:** A torn rotator cuff can cause persistent pain and discomfort in the shoulder, which can affect your ability to perform daily activities and interfere with sleep.
2. **Limited Range of Motion:** Over time, the lack of appropriate treatment can lead to further loss of shoulder motion and flexibility. This can make it challenging to reach, lift, or move the arm.
3. **Muscle Atrophy:** Without proper use and rehabilitation, the muscles around the shoulder can weaken and atrophy (shrink), leading to further loss of strength and function.
4. **Impaired Functionality:** As the tear progresses, it can impact your ability to perform routine tasks, such as reaching for objects, dressing, and grooming.
5. **Frozen Shoulder (Adhesive Capsulitis):** Untreated rotator cuff tears can contribute to the development of adhesive capsulitis, commonly known as frozen shoulder. This condition involves the thickening and tightening of the shoulder joint capsule, leading to pain and significant loss of shoulder mobility.
6. **Tendon Retraction and Irreparable Tears:** Over time, some rotator cuff tears can worsen and the torn tendons can retract (pull away from their attachment points). This can lead to more complex and potentially irreparable tears that are more difficult to surgically repair.
7. **Degenerative Changes:** Untreated tears can contribute to degenerative changes in the shoulder joint, including arthritis, which can further exacerbate pain and functional limitations.
8. **Chronic Inflammation:** The persistent inflammation associated with an untreated tear can contribute to long-term pain and discomfort.
9. **Secondary Shoulder Problems:** Compensation patterns developed due to the torn rotator cuff can lead to secondary shoulder problems, such as impingement or bursitis.

It's important to note that not all rotator cuff tears require surgical intervention, and some smaller tears can be managed effectively with conservative treatments such as physical therapy, activity modification, and pain management. However, it's generally recommended to seek medical evaluation and treatment for a suspected rotator cuff tear to prevent potential complications and ensure the best possible outcome for shoulder health and function. If you suspect you have a rotator cuff tear or are experiencing shoulder pain and dysfunction, it's advisable to consult with a healthcare professional.

Can you wait too long for rotator cuff surgery?

Yes, there is a point at which waiting too long for rotator cuff surgery can impact the potential success of the procedure and the overall outcome. The decision of when to have rotator cuff surgery is influenced by various factors, including the size and severity of the tear, your age, your activity level, and your overall shoulder health.

Waiting too long for surgery can lead to several potential issues:

1. **Tendon Quality and Repairability:** Over time, the torn tendon edges may retract and degenerate, making it more difficult for the surgeon to repair them. This can lead to a situation where the tear becomes irreparable or requires more complex surgical techniques.
2. **Muscle Atrophy and Weakness:** If the tear is left untreated for an extended period, the muscles around the shoulder joint can weaken and atrophy (shrink). This muscle loss can impact the success of the surgical repair and the overall strength and function of the shoulder after surgery.
3. **Limited Range of Motion:** Prolonged shoulder dysfunction can lead to a decrease in range of motion, making it more challenging to achieve optimal results even after surgery.
4. **Development of Secondary Problems:** Compensation patterns and altered biomechanics due to the untreated tear can contribute to the development of secondary shoulder problems, such as impingement, bursitis, or frozen shoulder.
5. **Chronic Pain and Discomfort:** Delaying surgery may result in ongoing pain, discomfort, and limited functionality that affect your quality of life.
6. **Increased Rehabilitation Challenges:** Longer periods of inactivity and muscle atrophy can lead to more challenging and prolonged post-operative rehabilitation.

However, it's also important to note that not all rotator cuff tears require immediate surgical intervention. Smaller tears and tears in individuals with lower activity levels may be managed successfully with conservative treatments such as physical therapy, activity modification, and pain management.

The decision of when to undergo rotator cuff surgery should be made in consultation with a qualified orthopedic surgeon who can assess your individual condition, discuss the potential risks and benefits of surgery, and provide personalized recommendations based on your situation. Early intervention can often lead to better surgical outcomes and improved long-term shoulder health.

Is rotator cuff surgery considered a major surgery ?

Yes, rotator cuff surgery is generally considered a major surgery. While it is typically performed using minimally invasive techniques such as arthroscopy, the surgery still involves repairing or reattaching torn tendons within the shoulder joint, which is a complex and delicate procedure. The rotator cuff is a group of four tendons and muscles that provide stability and movement to the shoulder joint. Surgery to repair a torn rotator cuff is often necessary to restore proper shoulder function and relieve pain.

Here are some reasons why rotator cuff surgery is considered a major procedure:

1. **Complex Anatomy:** The shoulder joint has intricate anatomy, and surgery involves working around important structures such as nerves, blood vessels, and other soft tissues.
2. **Surgical Skill:** The surgery requires a skilled orthopedic surgeon with expertise in shoulder anatomy and surgical techniques.
3. **Anesthesia:** While it's often performed under regional or local anesthesia, the procedure can still involve sedation or general anesthesia, which carries its own risks.
4. **Recovery and Rehabilitation:** Recovery from rotator cuff surgery involves a period of immobilization, followed by a structured rehabilitation program to regain strength, range of motion, and function.
5. **Potential Complications:** As with any surgery, there are potential risks and complications associated with rotator cuff surgery, such as infection, nerve injury, stiffness, and failure of the repair.
6. **Impact on Daily Life:** The recovery process and rehabilitation may significantly impact your daily activities and require adjustments to your lifestyle for a period of time.

It's important to note that the term "major surgery" doesn't necessarily refer to the size of the incision or the invasiveness of the procedure, but rather to the complexity of the surgery, the potential impact on your body, and the need for specialized medical care and rehabilitation. If you're considering rotator cuff surgery, it's essential to have a thorough discussion with your orthopedic surgeon to understand the procedure, potential risks, benefits, and the expected recovery process.

Why is rotator cuff surgery so painful?

Rotator cuff surgery can be painful for several reasons, including the nature of the procedure, the sensitivity of the shoulder joint, and the healing process that follows. Here are some factors that contribute to post-operative pain after rotator cuff surgery:

1. **Tissue Trauma:** Rotator cuff surgery involves repairing or reattaching torn tendons and muscles in the shoulder. The surgical manipulation and repositioning of tissues during the procedure can cause tissue trauma, which triggers pain signals.
2. **Inflammation and Swelling:** Surgery triggers an inflammatory response as part of the body's natural healing process. Inflammation and swelling in and around the surgical site can contribute to pain and discomfort.
3. **Nerve Irritation:** The surgical procedure, along with inflammation and swelling, can irritate nerves in the area, leading to pain. Nerves can be sensitive structures, and their involvement in the surgical process can contribute to post-operative discomfort.
4. **Muscle Spasm and Tension:** The muscles around the shoulder joint may experience spasm and tension in response to the surgical procedure and changes in biomechanics. Muscle tension and spasm can contribute to pain and limited range of motion.
5. **Recovery and Healing:** The healing process after surgery involves the repair and regeneration of tissues. During this period, the body goes through various stages of healing, which can be accompanied by pain and discomfort.
6. **Rest and Immobilization:** After surgery, the shoulder may be immobilized in a sling or brace to promote healing. Immobilization can lead to muscle stiffness, weakness, and discomfort.
7. **Physical Therapy:** While physical therapy is an important part of the recovery process, it can initially involve exercises and movements that may cause discomfort as the shoulder adapts to increased activity.
8. **Individual Pain Perception:** Pain tolerance and perception vary from person to person. Some individuals may experience more intense pain after surgery than others.

It's important to manage post-operative pain effectively to ensure a successful recovery. Your surgeon will provide guidance on pain management strategies, which may include pain medications, ice therapy, elevation, and gentle movement exercises. Following your surgeon's instructions and attending post-operative appointments are crucial for monitoring your recovery progress and addressing any concerns you may have about pain or discomfort. Over time, as healing progresses and you engage in rehabilitation, the pain should gradually diminish, and your shoulder function should improve.

How long does a rotator cuff repair last ?

The longevity of a rotator cuff repair can vary based on several factors, including the size of the tear, the surgical technique used, your adherence to rehabilitation, your overall health, and your activity level. A successful rotator cuff repair can provide long-lasting relief from pain and improve shoulder function, but it's important to understand that not all repairs are equal, and there is no definitive timeframe for how long a repair will last.

In some cases, a well-executed rotator cuff repair can last for many years, allowing individuals to resume their normal activities without significant pain or limitations. However, there are situations where the repaired tendon may experience degeneration or re-tear over time, especially in cases of larger or more complex tears.

Here are some factors that can influence the longevity of a rotator cuff repair:

1. **Tear Size and Location:** Larger tears or tears involving more critical areas of the rotator cuff may be more prone to re-tearing or degeneration over time.
2. **Surgical Technique:** The specific surgical technique used, as well as the skill of the surgeon, can impact the success and durability of the repair.
3. **Rehabilitation:** Adhering to a thorough and well-structured rehabilitation program is crucial for optimizing the healing process and maintaining the integrity of the repair.
4. **Age and Health:** Older individuals may have slower healing rates, and overall health can influence the body's ability to maintain tissue integrity.
5. **Activity Level:** High-demand activities or repetitive overhead motions can increase the risk of stressing the repaired tendon and potentially leading to re-injury.
6. **Smoking and Medical Conditions:** Smoking and certain medical conditions can impair the body's healing processes and affect the success of the repair.

It's important to follow your surgeon's post-operative instructions, attend all recommended follow-up appointments, and engage in prescribed rehabilitation exercises to maximize the chances of a successful and long-lasting rotator cuff repair. If you have concerns about your shoulder health or the longevity of a previous rotator cuff repair, it's advisable to consult with an orthopedic surgeon who can evaluate your individual situation and provide guidance based on your specific needs and goals.

Is rotator cuff surgery worth it ?

The decision of whether rotator cuff surgery is worth it depends on several factors, including the severity of your condition, the impact of the tear on your daily life and activities, your overall health, and your goals for recovery. Here are some considerations to help you make an informed decision:

1. **Pain and Function:** If your rotator cuff tear is causing significant pain, limiting your range of motion, and affecting your ability to perform daily activities, work, or recreational activities, surgery may be worth considering to alleviate these issues.
2. **Conservative Treatments:** Before opting for surgery, you may have tried conservative treatments such as physical therapy, rest, anti-inflammatory medications, and corticosteroid injections. If these treatments have not provided adequate relief and your symptoms persist, surgery may be worth exploring.
3. **Tear Size and Location:** The size and location of the tear can influence the potential benefits of surgery. Large or full-thickness tears may have a greater impact on shoulder function and may benefit more from surgical intervention.
4. **Goals and Lifestyle:** Consider your personal goals and lifestyle. If you have specific activities or sports that you want to return to, surgery may be a viable option to help you regain function and participation.
5. **Age and Health:** Your age and overall health can affect how well you recover from surgery. Younger, healthier individuals may have a better chance of successful recovery and long-term benefit.
6. **Risks and Complications:** Like any surgical procedure, rotator cuff surgery carries risks and potential complications. These should be discussed with your surgeon, and the potential benefits should be weighed against the risks.
7. **Recovery and Rehabilitation:** Rotator cuff surgery requires a period of recovery and rehabilitation, during which you will need to commit to following post-operative instructions and engaging in physical therapy. Your willingness and ability to undergo this process are important considerations.
8. **Patient Expectations:** Having realistic expectations about the outcomes of surgery is crucial. While surgery can often significantly improve shoulder function and alleviate pain, it may not always restore your shoulder to its pre-injury state.

It's important to have a thorough discussion with your orthopedic surgeon to fully understand your options, the potential benefits and risks of surgery, and what you can expect during the recovery process. Your surgeon can provide personalized recommendations based on your individual case and help you make an informed decision that aligns with your goals and priorities. If you're unsure about whether surgery is the right choice for you, consider seeking a second opinion to gather additional perspectives on your condition and treatment options.

How can I speed up recovery after rotator cuff surgery ?

Recovery after rotator cuff surgery requires a combination of proper care, adherence to post-operative instructions, and a well-structured rehabilitation program. While everyone's recovery timeline is unique, there are several steps you can take to potentially speed up your recovery and optimize your shoulder's healing process. It's important to note that pushing yourself too hard or trying to speed up recovery too quickly can lead to complications, so it's crucial to follow your surgeon's guidance and recommendations. Here are some tips to consider:

1. **Follow Post-Operative Instructions:** Your surgeon will provide specific instructions for wound care, immobilization, and early activities. Follow these instructions closely to ensure proper healing.
2. **Manage Pain and Swelling:** Proper pain management is essential for a comfortable recovery. Take prescribed pain medications as directed, and use ice packs (wrapped in a cloth) to reduce swelling in the early days after surgery.
3. **Begin Rehabilitation Early:** Physical therapy is a key component of your recovery. Start your prescribed rehabilitation exercises as soon as recommended by your surgeon or physical therapist. These exercises will help improve range of motion and strengthen the muscles around your shoulder.
4. **Attend Physical Therapy:** Attend all scheduled physical therapy sessions and follow your therapist's guidance. They will gradually increase the intensity of exercises to help you regain strength, flexibility, and function.
5. **Maintain a Healthy Diet:** Eating a balanced diet rich in nutrients can support the healing process. Adequate protein intake is particularly important for tissue repair.
6. **Stay Hydrated:** Drinking enough water is essential for overall health and can aid in tissue healing and recovery.
7. **Rest and Sleep:** Give your body sufficient rest and sleep to aid in the healing process. Avoid overexertion and ensure you're getting quality sleep each night.
8. **Use Supportive Equipment:** If your surgeon recommends it, use a sling or brace as directed to support and protect your shoulder during the initial healing phase.
9. **Gradually Increase Activities:** As you progress in your recovery, gradually reintroduce normal daily activities and light tasks. Avoid heavy lifting and strenuous activities until cleared by your surgeon.
10. **Communication with Your Healthcare Team:** Maintain open communication with your surgeon and physical therapist. Report any unusual symptoms, concerns, or changes in your condition promptly.
11. **Avoid Smoking and Alcohol:** If you smoke, consider quitting or at least reducing smoking during your recovery period. Smoking can impair the healing process. Similarly, alcohol consumption can interfere with healing and pain management, so it's best to avoid excessive drinking.
12. **Stay Positive and Patient:** Recovery takes time, and there may be ups and downs along the way. Staying positive, patient, and committed to your rehabilitation will contribute to a successful outcome.

Remember that each person's recovery is unique, and it's important to listen to your body and progress at a pace that is safe and comfortable. Always consult with your surgeon and healthcare team before making any significant changes to your recovery plan.
